# Detecting Acute Otitis Media Symptom Episodes Using a Mobile App: Cohort Study

**DOI:** 10.2196/mhealth.7505

**Published:** 2017-11-28

**Authors:** Annemarijn C Prins-van Ginkel, Marieke LA de Hoog, C Uiterwaal, Henriette A Smit, Patricia CJ Bruijning-Verhagen

**Affiliations:** ^1^ Julius Center for Health Sciences and Primary Care University Medical Center Utrecht Utrecht Netherlands

**Keywords:** smartphone, mobile app, infectious diseases, cohort studies, acute otitis media, underreporting, patient compliance, mobile applications, communicable diseases, otitis media

## Abstract

**Background:**

Population cohort studies are useful to study infectious diseases episodes not attended by health care services, but conventional paper diaries and questionnaires to capture cases are prone to noncompliance and recall bias. Use of smart technology in this setting may improve case finding.

**Objective:**

The objective of our study was to validate an interactive mobile app for monitoring occurrence of acute infectious diseases episodes in individuals, independent of health care seeking, using acute otitis media (AOM) symptom episodes in infants as a case study. We were interested in determining participant compliance and app performance in detecting and ascertaining (parent-reported) AOM symptom episodes with this novel tool compared with traditional methods used for monitoring study participants.

**Methods:**

We tested the InfectieApp research app to detect AOM symptom episodes. In 2013, we followed 155 children aged 0 to 3 years for 4 months. Parents recorded the presence of AOM symptoms in a paper diary for 4 consecutive months and completed additional disease questionnaires when AOM symptoms were present. In 2015 in a similar cohort of 69 children, parents used an AOM diary and questionnaire app instead.

**Results:**

During conventional and app-based recording, 93.13% (17,244/18,516) and 94.56% (7438/7866) of symptom diaries were returned, respectively, and at least one symptom was recorded for 32.50% (n=5606) and 43.99% (n=3272) of diary days (*P*<.01). The incidence of AOM symptom episodes was 605 and 835 per 1000 child-years, respectively. Disease questionnaires were completed for 59% (17/29) of episodes when participants were using conventional recording, compared with 100% (18/18) for app-based recording.

**Conclusions:**

The use of the study’s smart diary app improved AOM case finding and disease questionnaire completeness. For common infectious diseases that often remain undetected by health care services, use of this technology can substantially improve the accurateness of disease burden estimates.

## Introduction

A key issue in many prospective infectious disease epidemiological studies, both interventional and observational, is completeness of detecting disease events of interest among study participants. This is particularly true for events that cannot be comprehensively and reliably detected through health care-based research, such as self-limiting respiratory or gastrointestinal infections. For instance, in the Netherlands, it is estimated that health care encounters are not involved for up to 50% of acute otitis media (AOM) episodes in young children. AOM is therefore notoriously underdetected when health care contacts alone are relied on [1-3]. There is evidence that AOM poses a substantial burden, both on the child and the family, and results in economic losses due to workdays lost irrespective of health care use, thus requiring assessment of participant-reported disease events when population disease burden is estimated [1,4,5]. Moreover, health-seeking behavior is influenced by factors other than severity and could therefore introduce bias. In the field of infectious diseases epidemiology, this forms a major challenge in conducting research. For these reasons, there is a great interest in methods that can improve detection of infectious diseases events in epidemiological studies beyond the current health care-related scope.

Research on mobile apps for monitoring or promoting patient health is expanding rapidly, forming an entire new area within epidemiology. Most research focuses on evaluations of mHealth apps as an intervention in which apps are used to improve health [6,7]. However, well-designed mobile apps can also serve as valuable instruments to document health and disease among study participants. Up to now, apps have been little used as an alternative data-recording tool, while this technology offers several advantages over traditional methods of participant follow-up, such as paper, Internet, or telephone surveys. Interactive and dynamic features can be applied to improve participant compliance, detection, and disease burden estimation [7,8].

We performed a proof-of-concept validation study of an interactive mobile app for monitoring occurrence of acute infectious diseases episodes in individuals, independent of health care seeking, using AOM symptom episodes in infants as a case study. Our aim was to determine participant compliance and app performance in detecting and ascertaining (parent-reported) AOM symptom episodes with this novel tool compared with traditional methods used for monitoring study participants.

## Methods

We compared use of traditional survey methods (paper diary sheets) with use of a smartphone diary app for the purpose of prospectively detecting AOM symptom episodes and measuring their disease burden in infants. We made comparisons by applying the different methods consecutively over 2 periods (in 2013 and 2015) of a nested AOM study within a larger ongoing birth cohort study.

### WHISTLER Study

**Figure 1:**
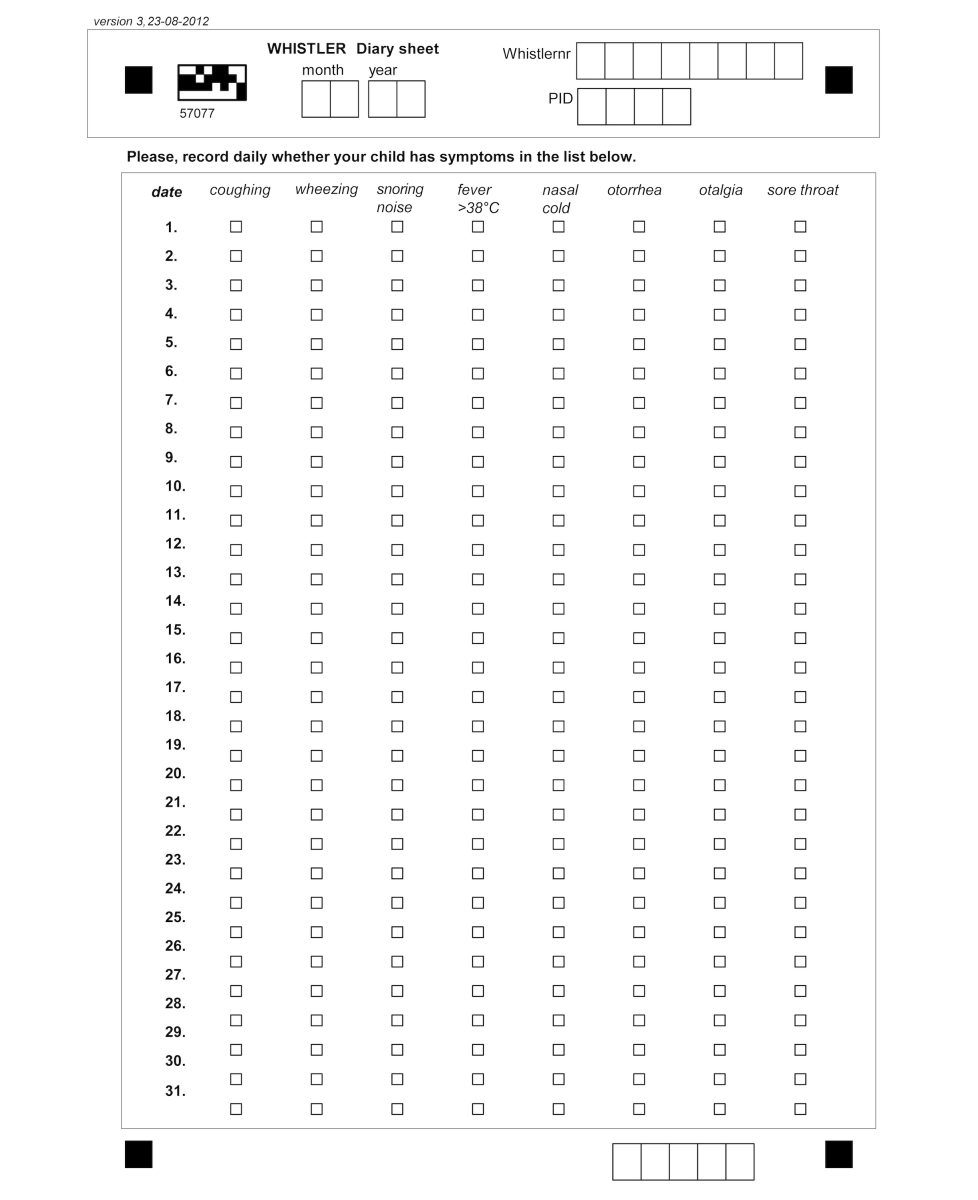
Paper diary sheet used in the 2013 study period. WHISTLER: Wheezing and Illnesses Study Leidsche Rijn.

Both the first (2013) and second (2015) AOM study period were (partly) nested within the Wheezing and Illnesses Study Leidsche Rijn (WHISTLER) birth cohort that recruited healthy, term neonates between 2001 and 2012 to study perinatal and infant risk factors for wheezing illness. The study design and rationale of WHISTLER are described in detail elsewhere [9]. WHISTLER enrolled newborns before 2 months of age and living in the Leidsche Rijn district of Utrecht, the Netherlands. WHISTLER had traditionally been using paper diary sheets to monitor parent-reported respiratory symptoms during the first year of life. For this purpose, a diary sheet containing a list of respiratory symptoms printed for each day of the month was distributed monthly by mail. Symptoms included cough, wheeze, fever (>38°C), nasal cold, otorrhea, otalgia, and sore throat (Figure 1). Parents were asked to mark whenever a symptom was present on a particular day. Additionally, the diary sheet contained a monthly questionnaire about environmental risk factors of respiratory infections printed on the back. After the end of each month, parents mailed the filled-in paper sheet in return envelopes, and the sheets were scanned for digital processing [9].

### Design of the 2013 Acute Otitis Media Study Period

In January 2013, we invited by mail 300 participating WHISTLER parents (randomly selected out of 594 parents) with children aged 0 to 3 years for additional participation in our nested AOM study. According to the WHISTLER traditional method, parents reported respiratory symptoms and answered the monthly questionnaire using the paper diary sheets. For children older than 1 year, we requested parents to restart recording during 4 consecutive months (February to May 2013), as symptoms were recorded only in the first year of life according to the WHISTLER protocol. In addition to the daily recording, parents were asked to contact the study team within 24 hours by email, text message, or telephone call when they recorded a combination of symptoms suggestive of an AOM symptom episode. For this, parents received detailed instructions upon enrollment explaining which (combination of) symptoms was suggestive of an AOM symptom episode and should prompt notification. Researchers verified these symptoms over the telephone. Subsequently, we asked parents to complete paper versions of a validated AOM severity score (AOM-SOS) during 7 consecutive days and an additional disease questionnaire on day 7 [10]. Questionnaires and scoring lists were returned by mail. We used these to ascertain the AOM symptom episode by assessing AOM severity, health care use, and impact on family life.

### Design of the 2015 Acute Otitis Media Study Period

We invited 404 parents of 0- to 3-year-old children by mail to participate in the (nested) AOM study. As WHISTLER completed recruitment in January 2013, no infants under 1 year of age were participating in WHISTLER in January 2015. Thus, we invited 91 WHISTLER age-eligible (ie, <3 years) participants and an additional 313 non-WHISTLER participants aged between 3 months and 2 years who also lived in the Leidsche Rijn district. Instead of using the paper diary sheet, all participating parents were now instructed to use a mobile device diary app, the InfectieApp, which we developed for this study, during 4 consecutive months (February to May 2015).

During each study period, we used identical criteria for occurrence of an AOM symptom episode: a combination of fever and either otalgia or otorrhea on the same day. This combination of symptoms had to be actively reported by the parents in the 2013 pilot study, while this combination was automatically recognized and reported using the InfectieApp in the 2015 pilot study.

Each period of the AOM study received separate approval by the medical ethics committee of the University Medical Centre Utrecht. Written informed consent was given by all participating parents.

### InfectieApp

**Figure 2:**
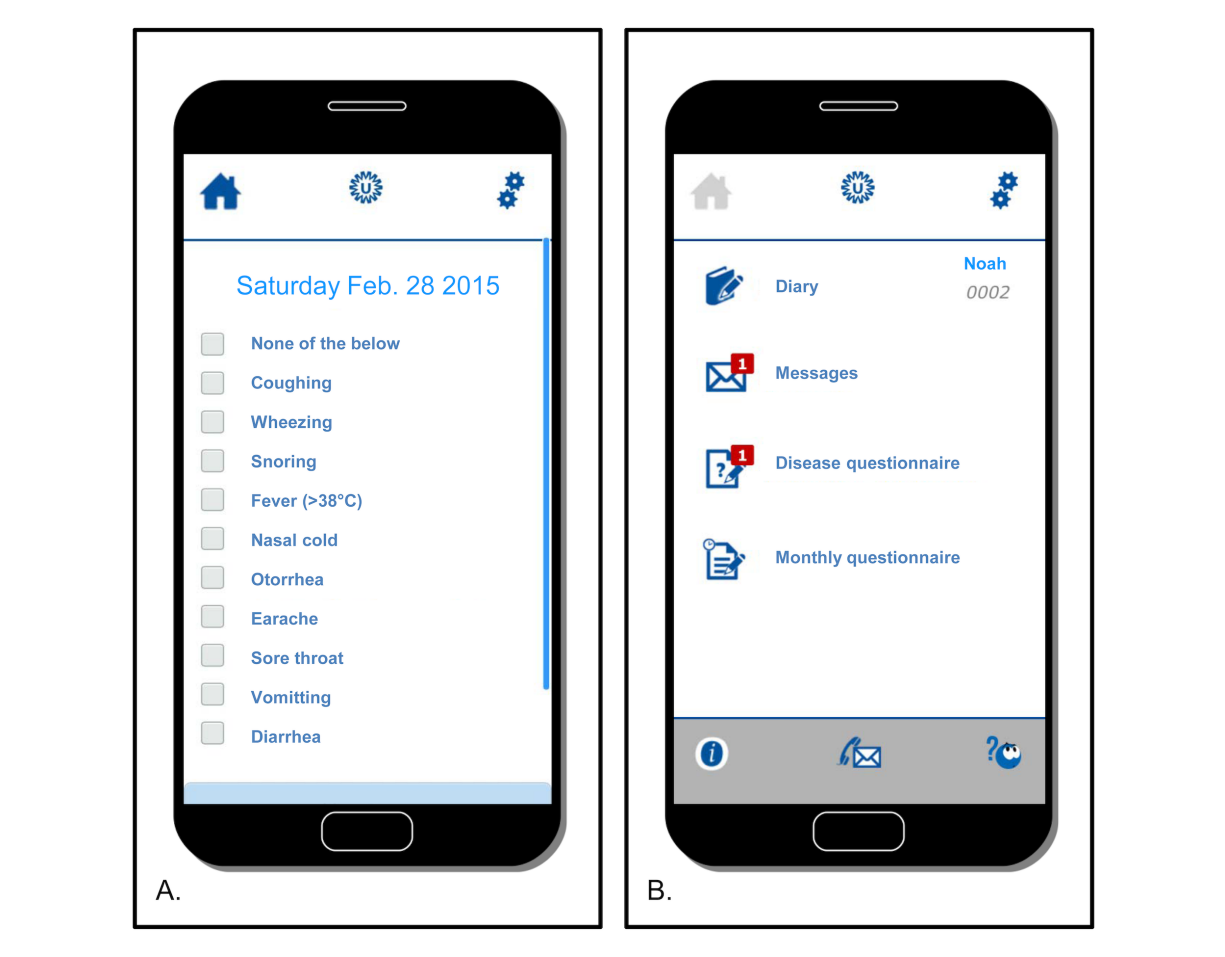
Screenshots of the diary app (InfectieApp) used in the 2015 study period. (A) symptom diary; (B) home screen.

The InfectieApp software app was custom made in 2014 by University Medical Centre Utrecht in collaboration with VitalHealth Solutions, a company specializing in eHealth, based in Uddel, the Netherlands, and was compatible with iOS, Android, and Windows Mobile operating systems. The InfectieApp was developed for use by study participants to self-report 3 types of data: (1) symptom diary data (Figure 2, panel A), (2) monthly questionnaire responses on risk factors, and (3) disease questionnaire responses and scoring lists in the event of an AOM symptom episode. The symptoms listed in the app diary, disease questionnaire, and scoring list were identical to those in the questionnaires used in the 2013 study period. Participants could access additional study-related information in the app organized in various submenus, including general patient information related to the study, frequently asked questions, and contact details of the research team. All app content was organized in menus, accessible through the home screen (Figure 2, panel B).

The onset and ending of an AOM symptom episode was detected based on diary entries, using built-in algorithms: fever together with either earache or otorrhea occurring on the same day marked the onset of an episode. An episode ended when fever was not recorded for 7 consecutive days. Detection of a new-onset AOM episode triggered additional app content: participants received an app message explaining that AOM symptoms were detected and that additional questionnaires would follow in the coming days. For ascertainment of the AOM symptom episodes, the AOM-SOS scoring list and disease questionnaire appeared in the app questionnaire menu. The message also contained a link to a Dutch independent, professional, patient website on AOM where parents could read general AOM medical information [11-13].

To encourage participant compliance with diary recording, daily reminders at 8 PM appeared as push notifications on the smartphone. A diary that had not been filled in remained accessible to the participant up to 7 days after the diary date. We could also decide to contact the household by telephone.

The InfectieApp was password protected at first log-in; thereafter, the participant could use a 4-digit code to enter the app. When the app was used offline, data were stored encrypted in the InfectieApp. The recorded data were sent to the server via a secure connection (hypertext transfer protocol secure, HTTPS), meaning that the data were sent encrypted to the server. When the app was online, the recorded data were sent immediately to the server, providing us with the opportunity to monitor the participants in real time both for the occurrence of AOM symptom episodes and for compliance with the questionnaires.

A security access layer determined which actions a user could perform, meaning that, depending on the rights assigned to the user, the user could or could not perform certain actions. This server was hosted by VitalHealth Solutions. The research team could access the study data stored on the server using a Web interface. Each researcher had access to the decrypted data using their personal username and passwords.

### Data Analysis

For the primary outcome, we compared the proportion of AOM symptom episodes ascertained by complete disease questionnaires between both study periods. Next, we compared the number of diaries completed and the number of monthly questionnaires filled in. For the 2015 study period, we compared the difference in number of diaries and monthly questionnaires completed between the WHISTLER and non-WHISTLER participants.

In the 2015 study period, we could assess the number of diaries completed more reliably, because a confirmation of absence of symptoms was required (symptom checkbox “None of the below”; Figure 2, panel A), while no such checkbox existed on the paper diary sheet. We therefore decided to assess and compare diary completeness using 2 different outcomes. First, we compared the proportion of diaries returned by mail (first period) with the proportion entered using the app (second period). For this, we assumed that, for a returned paper diary sheet, each day of the month was completed, although a day without a reported symptom could represent a missing day. Second, we compared the proportion of days with at least one reported symptom in both study periods, thereby automatically excluding potentially missing days on diary sheets in the first study period.

We estimated the incidence rate of AOM symptom episodes as the number of AOM symptom episodes per 1000 child-years and compared the 2 study periods by using OpenEpi (Open Source Epidemiologic Statistics for Public Health, version 3.01 [14]). For the 2013 study period, we based the incidence on the number of paper diaries returned.

The supplementary analysis included an assessment of characteristics of the ascertained AOM symptom episodes in both periods. We compared AOM-SOSs, as well as other characteristics, including health care use, medication, and parental work absenteeism derived from the disease questionnaire.

Comparisons were made using chi-square and independent-sample *t* tests, where appropriate. Calculations were conducted using IBM SPSS version 22.0 (IBM Corporation). A *P* value of <.05 was considered statistically significant.

## Results

### Study Population

**Figure 3:**
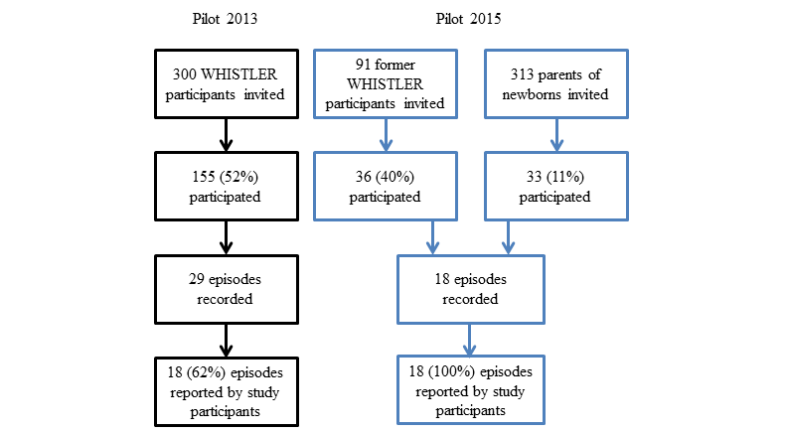
Flowcharts of the study population. WHISTLER: Wheezing and Illnesses Study Leidsche Rijn.

Of the 300 invited WHISTLER participants, 155 (51.7%) participated during the first study period (2013) and returned at least one paper diary sheet. For the second study period (2015), 69 (17.1%) of 404 invited parents participated and completed at least one monthly app questionnaire. Of these, 36 (52%) were former WHISTLER participants (Figure 3) and of whom 11 (16%) had also participated in the 2013 study period. The baseline participant characteristics were comparable during both study periods (Table 1).

**Table 1:** Baseline characteristics of the 2013 and 2015 study populations.

Characteristics			Study				*P* value
			2013 paper diary (n=155)		2015 diary app (n=69)		
**Infant characteristics**							
	Age (years), mean (SD)	1.50 (0.72)		1.76 (0.78)		.02	
	Male sex, n (%)	69 (45)		32 (46)		.88	
	Participant with at least 1 sibling, n (%)	93 (60)		29 (54)		.43	
	Daycare visit during study months^a^, n (%)	138 (89)		55 (80)		.09	
	Former WHISTLER^b^ study participant, n (%)	155 (100)		36 (52)		<.001	
	≥1 AOM^c^ symptom episode, n (%)	24 (16)		13 (19)		.56	
**Parent characteristics**							
	Age (years), mean (SD)	36.2 (3.95)		35.7 (3.75)		.38	
	High level of education^d^, n (%)	131 (89)		60 (92)		.47	

^a^Minimum of 1 month of daycare during study period.

^b^WHISTLER: Wheezing and Illnesses Study Leidsche Rijn.

^c^AOM: acute otitis media.

^d^Defined as 1 or both parents having a high vocational or university degree.

### Questionnaire Completeness

In 29 diary sheets of 24 different children, the criteria for an AOM symptom episode (fever in combination with otalgia or otorrhea) were met in the 2013 study period, and 18 (62%) of these episodes were actively reported to the study team by the parents. For 17 (59%) episodes, an AOM-SOS scale was completed for 7 days by the parents, and 15 (52%) disease questionnaires were completed. In the 2015 study period, the app automatically detected 18 AOM symptom episodes in 13 different children. For all (100%) of these episodes, the parents completed the questionnaire about health care use and family impact, and 7 days of the AOM-SOS scale. Ascertainment of AOM symptom episodes (*P*=.003) and completeness of AOM reporting (*P*=.003), including AOM-SOS and disease questionnaire (*P*=.001), were significantly higher in the 2015 study period (Table 2).

The 2013 study period contained a total of 18,516 observation days. Data were received for 17,244 days (93.13%). In 2015, data were recorded by the app for 7438 of the 7866 observation days (94.56%, *P*<.001 for difference in number of recorded observation days between the 2 study periods; Table 2). Of the 17,244 reported days in 2013, symptoms were recorded for 5605 (32.50%) as compared with 3272 symptom days out of 7438 days (43.99%) in the 2015 study period (*P*<.001).

During the 2013 study period, of the 617 monthly questionnaires that could have been completed, 575 (93.2%) were returned to the study team. In the 2015 study period, 299 of 329 (90.9%) monthly questionnaires were completed (*P*=.20).

During the 2015 study period, for the former WHISTLER participants, data were retrieved for 4090 of the 4271 observation days (95.8%), while for the non-WHISTLER participants, data were retrieved for 3348 of the 3595 observation days (93.1%) (*P*<.001). The percentage of completed monthly questionnaires was not significantly different between the former WHISTLER participants and the newly recruited parents of newborns in the 2015 study period (*P*=.13).

The incidence of AOM symptom episodes was 605 per 1000 child-years in 2013 and 835 per 1000 child-years in 2015 (Table 2). Table 3 shows characteristics and AOM-SOSs of AOM symptom episodes in both study periods. There were no significant differences in characteristics and AOM-SOSs of ascertained AOM symptom episodes between study periods.

**Table 2:** Acute otitis media (AOM) incidence and participant compliance with study procedures.

Questionnaire results		Study period		*P* value
		2013	2015	
**AOM incidence**				
	AOM symptom episodes, n	29	18	.003
	AOM incidence/1000 child-years	605	835	.29
	AOM-SOS^a^ questionnaires completed, n (%)	17 (59)	18 (100)	.003
	Disease questionnaire completed, n (%)	15 (52)	18 (100)	.001
**Participant compliance with recording^b^**				
	Total days for which data received, n/N (%)	17,244/18,516 (93)	7438/7866 (95)	<.001
	Total days with ≥1 symptom reported in diary, n (%)	5605 (33)	3272 (44)	<.001
	Monthly questionnaires completed, n/N (%)	575/617 (93)	299/329 (91)	.20

^a^AOM-SOS: acute otitis media severity score.

^b^The degree of compliance was compared between all participants of the 2013 and 2015 study periods (n=155 vs n=69).

**Table 3:** Characteristics of parent-reported acute otitis media symptom episodes.

Characteristics	Study period	
	2013	2015
Episodes with otalgia, n/N (%)	26/29 (90)	17/18 (94)
Episodes with otorrhea, n/N (%)	11/29 (38)	6/18 (33)
Number of days with fever, median (range)	3.0 (1-11)	2.0 (1-5)
Episodes for which parents stayed home, n/N (%)	6/15 (40)	9/18 (50)
Episodes when parents worried regularly to a lot, n/N (%)	7/15 (47)	9/18 (50)
Episodes for which antibiotics were prescribed, n/N (%)	5/15 (33)	6/18 (33)
General practitioner visits, n/N (%)	9/15 (60)	8/18 (44)
Highest AOM-SOS^a^, mean (SD)	8.6 (3.0)	9.9 (3.4)

^a^AOM-SOS: acute otitis media severity score. Highest possible AOM-SOS is 14 for each day. This score consists of 7 discrete items: tugging of ears, crying, irritability, difficulty in sleeping, diminished activity, diminished appetite, and fever. Parents were asked to rate these symptoms daily during 7 days following symptom onset in comparison with the child’s usual state, as “none,” “a little,” or “a lot,” with corresponding scores of 0, 1, and 2. Higher scores indicated more severe symptoms. For this study the AOM-SOS scale was translated into Dutch [10].

## Discussion

This study evaluated the use of a symptom diary app to detect the occurrence of parent-reported AOM in comparison with conventional (paper) survey methods accompanied by written instructions. The results of this study showed that improved case finding and completeness of disease burden information can be achieved by using an interactive app. Moreover, participants stayed well engaged with app procedures, resulting in 95% completeness of diary data.

Epidemiological research on common infectious diseases often struggles with underdetection of disease events [1-3]. This poses a threat to validity of incidence estimates and quantification of disease burden. For measurement of population incidence and disease burden of numerous infectious diseases, the use of interactive diary apps such as the prototype evaluated in this study could substantially improve accurateness of the estimates. Similarly, diary apps could be valuable in vaccine studies that typically require comprehensive detection and ascertainment of both disease events and potential vaccine side effects [15]. For such purposes, our prototype research app could be easily expanded to include tasks such as specimen collection. The study of Quee et al expanded the app with specimen collection and yielded similar high compliance rates for the questionnaires to those in this study, with 97% diary completeness and 93% to 98% completeness for the 3 different disease questionnaires used in Quee’s study (FA Quee, email communication, November 2016) [16].

The field of mobile apps health research (mHealth) is rapidly expanding [7]. Studies reporting on mHealth apps, applied as interventions to improve patient self-management or enhance patient monitoring and disease management, are numerous [17-29]. However, very little has been published on the value of apps as methodological tools in health-related human participant research. To our knowledge, our study is the first to evaluate the use of an interactive symptom diary app as a research tool for detection of infectious disease events that are difficult to capture by health care-based research. We could identify only 1 previous methodological study that compared the use of a diary app versus a paper diary for the reporting of pain-related symptoms. The use of the pain diary app increased completeness of records by approximately 34% [8]. While we evaluated the value of this diary app in epidemiological research, its application in clinical care to closely monitor a patient’s health and aid disease management could be a valuable expansion of the app.

The increasing number of smartphone users, the fact that most smartphone owners have their smartphone on or near them most of the day, and the computer features of smartphones make them attractive tools for health care and research [6,30,31]. The use of apps compared with conventional survey methods, such as paper- or Web-based questionnaires, improves usability for the end user because of the opportunity to include dynamic content and decreased administrative handling [8,31-33]. Moreover, mobile technology offers instant data transfer, allowing for real-time monitoring of and interaction with study participants [15,31]. Several studies have confirmed that study participants prefer the use of apps over paper versions or Web-based questionnaires [8,31,33-38]. While we did not systematically assess parental preferences in our study, the 2015 parents who were former WHISTLER participants, and had thus used paper diary sheets in the past, mentioned a clear preference for the symptom diary app. Combined, these advantages create a potential for increased response and study compliance, improved case detection, and reduced costs when apps are used.

One possible threat when using apps for epidemiological research is the possibility of introducing selection bias because of the requirement of smartphone ownership. In the Netherlands the use of smartphones is widespread and steadily increasing, especially in the age group of young parents, where it now reaches almost 90% [39]. Therefore, introducing apps as research tool does not create selection bias in this particular setting [39,40]. However, when designing a study, this should always be evaluated upfront, especially in countries with lower smartphone penetration or for elderly populations. Concerns have also been raised about the quality of the response when using apps instead of conventional paper questionnaires. However, a recent systematic review by Marcano Belisario et al confirmed that there were no quality differences between data collected via tablet or smartphone apps and paper questionnaires [31]. Another possible limitation of our study is the difference in participants included in the study periods. In 2013, only WHISTLER participants were included, while 2015 included both WHISTLER participants and newly recruited participants. Both the 2013 and the 2015 WHISTLER participants consented to the AOM study as an add-on to the main WHISTLER study and were probably a relatively committed subset. This may explain the somewhat different percentages of completed questionnaires for WHISTLER and non-WHISTLER participants in the 2015 period, where the latter had a lower response rate. Inclusion of newly recruited parents in the 2015 study period might therefore have resulted in an underestimation of the compliance difference and thus the added value of app-based monitoring. Also, 11 parents participated in both study periods. The fact that these parents consented to participate in both study periods, in addition to the WHISTLER study, probably reflects their commitment to scientific research, which was also reflected in the high response rates for both study periods. By analyzing the 2 cohorts as being independent, we may have underestimated the effect of diary app use on the completion rate, since the 11 participants had little room for improvement due to app use.

In conclusion, our results indicate that intensive follow-up of study participants by means of an interactive app has the potential to improve the data quality of infectious diseases occurrence in populations, especially for health events that are difficult to capture by health care-based research. Our findings could have important implications for design and execution of research, both observational and interventional, involving population disease burden quantification, especially when conducted in populations with a high percentage of smartphone users, because digitization will continue and, over time, paper questionnaires may become less accepted.

## Abbreviations

AOM: acute otitis media
AOM-SOS: severity score
